# Low-Grade Malignant Triton Tumor of the Neck: A Case Report and Review of the Literature

**DOI:** 10.1155/2014/674094

**Published:** 2014-09-22

**Authors:** Taissir Omar, Hanaa Raslan, Sahar El Sheikh, Moataz Rizq, Awatef Draz

**Affiliations:** ^1^Oral Pathology Department, Faculty of Dentistry, Alexandria University, Champollion Street, Azarita, Alexandria 21526, Egypt; ^2^Maxillofacial and Plastic Surgery Department, Faculty of Dentistry, Alexandria University, Champollion Street, Azarita, Alexandria 21526, Egypt; ^3^Oral Pathology Department, Faculty of Dentistry, King Abdulaziz University, P.O. Box 80200, Zip Code 21589, Jeddah 22252, Saudi Arabia; ^4^Cairo University, 12 EL-Saraya Street, Manial, Cairo 11553, Egypt

## Abstract

Rhabdomyoblastic differentiation in a malignant peripheral nerve sheath tumor (MPNST) is termed malignant triton tumor (MTT), a rare neoplasm that poses a diagnostic dilemma in the differential diagnosis of neck masses and portends poor prognosis. We report a sporadic case of MTT of the neck in a 23-year-old female. We present the pathological findings. Immunohistochemistry confirmed the neurogenic origin with S-100 expression and the rhabdomyoblastic differentiation with desmin and vimentin positivity. Radical surgical excision was done. After 4 years there were no signs of recurrence or distant metastasis. The clinical, microscopic, and long-term follow-up of this case are consistent with those of a low-grade malignancy.

## 1. Introduction

Malignant peripheral nerve sheath tumor (MPNST) is a rare soft tissue neoplasm with a poor prognosis. It can occur in association with neurofibromatosis type 1 (NF-1) or sporadically accounting for 5–10% of soft tissue sarcomas [1–4]. MPNST occurs in about 2–5% of patients with NF-1, compared with a prevalence of 0.001% in the general population [5, 6]. MPNST comprises only 2–6% of head and neck sarcomas [7]. The diagnosis, management, and histogenesis of MPNST continue to challenge pathologists and surgeons especially in sporadic cases [3, 8]. These tumors are thought to arise from Schwann cells, their precursors, or a preexisting NF-1 [4]. MPNST may consist of tissues such as glandular epithelium, squamous cells, cartilage, bone, or even adipose tissue. Tumors with skeletal muscle differentiation and malignant Schwann cells are classified as a subtype or a histologic variant of MPNST [9, 10] and are referred to as malignant triton tumor (MTT) [11]. This tumor was originally described by Masson in 1932. It is very rare and has a more aggressive clinical course and worse prognosis than MPNST without rhabdomyoblastic differentiation [12–14]. It was reported that the 5-year survival rate is 5–15% [13, 14], compared to 50–60% for MPNST [15].

In the majority of reported cases, triton tumors are located across peripheral nerves, usually close to the spine, in the head and neck region, or in the upper and lower extremities [16]. However, an intracardiac presentation has been reported within the atrium [17]. Wong et al. [18] reported that MTT involving nonextremity sites has a worse prognosis. In fact, the location, large size, and tumor stage are found to affect survival [15, 18]. The metastatic rate in patients with MTT is reported to be 31.4% [13]. Shorter survival was reported in cases of MTT not associated with NF-1 [11].

Two theories were proposed as an explanation to the development of this tumor [19]. The first was that Schwann cells in neurogenic tumor could be stimulated by motor nerve to differentiate into rhabdomyoblastic component. The second explanation was more plausible, where neoplastic Schwann cells can transform into rhabdomyoblasts, suggesting the possibility of some mesenchymal tissue derivation from neuroectodermal cells. Another opinion suggested a differentiation-metaplasia capacity of neuroectodermal tissue [20].

To further elucidate the natural history and prognosis of this rare neoplasm in the head and neck, we present a case of sporadic MTT arising in the neck with an unusual prognosis.

## 2. Case Presentation

A 23-year-old female patient presented to the craniomaxillofacial and plastic surgery department at our institution with a 10-month left neck swelling. Her medical and family histories were unremarkable and negative for prior radiation exposure and NF-1, respectively. Physical examination revealed a slightly tender, firm mass, fixed to the underlying structures, with a normal overlying skin and no palpable lymphadenopathy.

**Figure 1:**
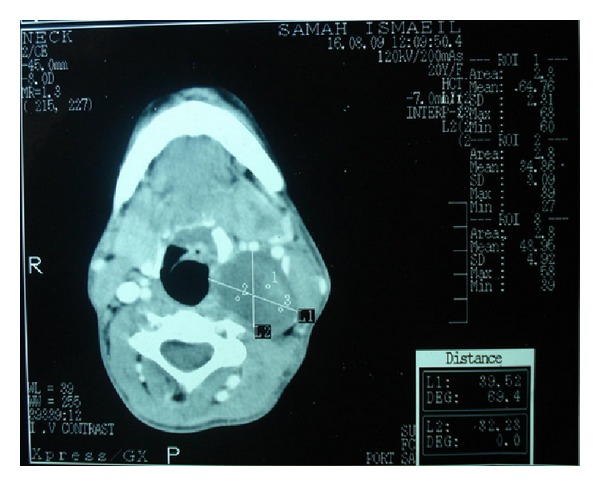
Axial view of CT showing dimensions of the tumor.

**Figure 2:**
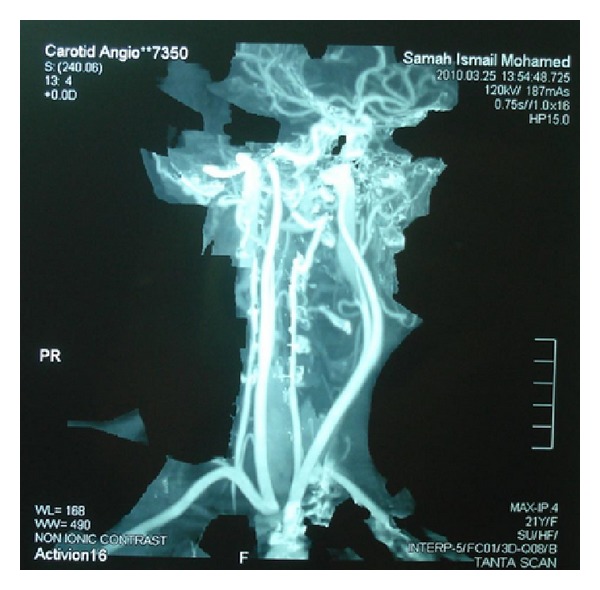
Coronal view of CT angiography showing left parapharyngeal and pharyngeal mainly hypodense, peripherally hyperdense well-defined lesion (7 × 4 × 3 cm) indenting the left pharyngeal wall but not infiltrating it.

Conventional computed tomography (CT) (Figure 1) and CT angiography (Figure 2) demonstrated a left parapharyngeal mass 3 × 4 × 9 cm, displacing the left internal carotid artery, external carotid artery, and internal jugular vein, with no evidence of vascular infiltration. An incisional biopsy revealed the diagnosis of sporadic-type MTT (de novo) of the neck. This was followed by a wide surgical excision of the mass under general anesthesia.

**Figure 3:**
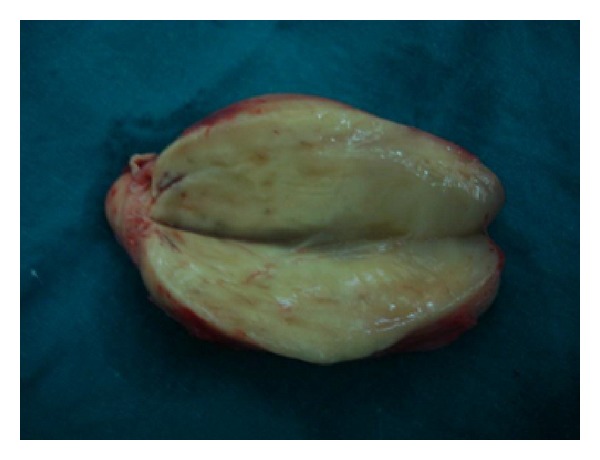
Cross-sectional image of the excised tumor.

**Figure 4:**
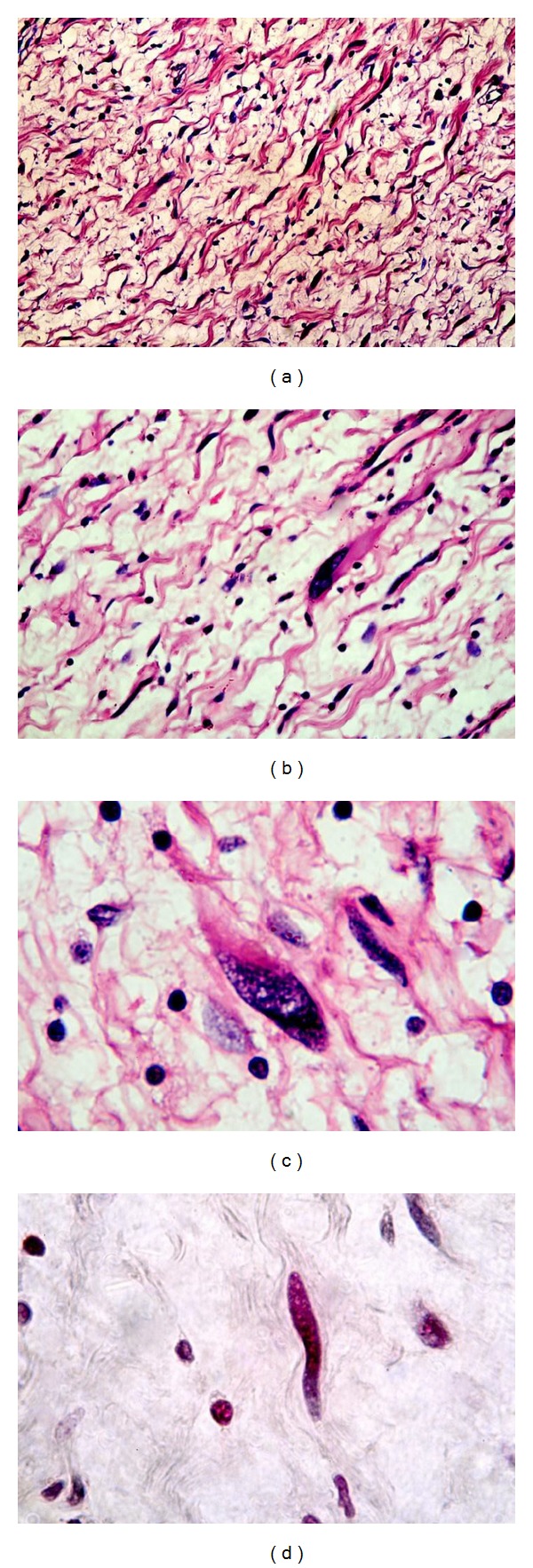
Malignant triton tumor showing the following: (a) well delineated spindle cells with scattered rhabdomyoblasts (H&E ×100), ((b) and (c)) higher magnification (×200 and ×400), and (d) cross striations of rhabdomyoblast cells stained with phosphotungstic acid hematoxylin (×400).

**Figure 5:**
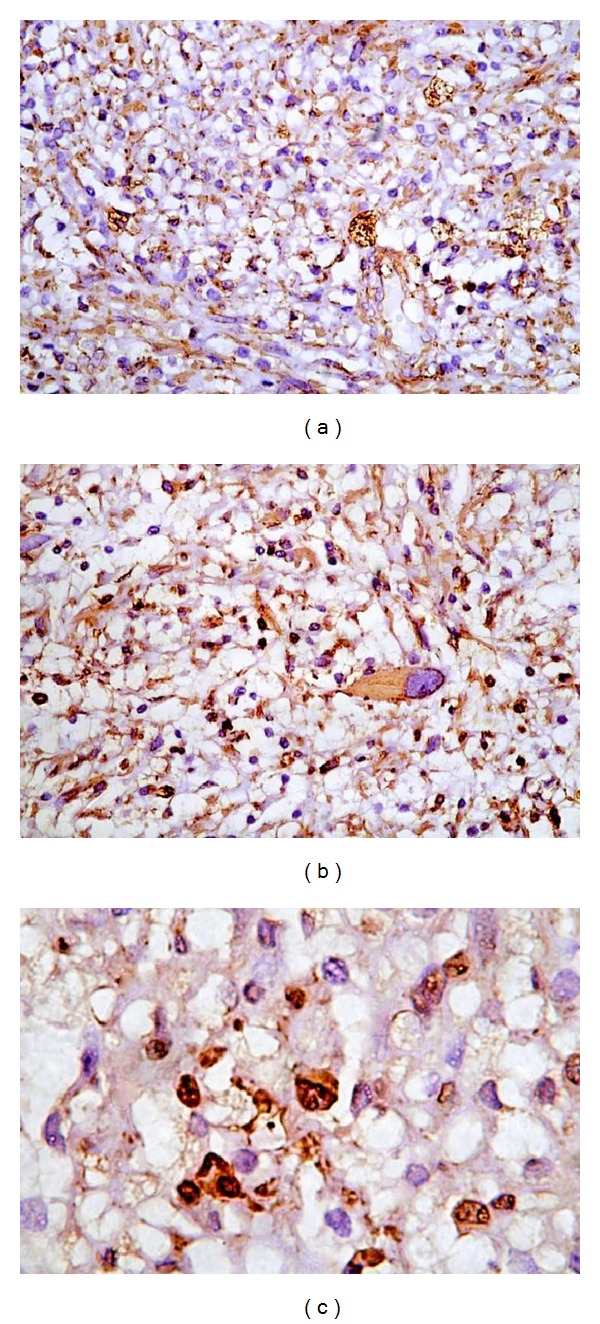
Immunohistochemical staining of malignant triton tumor showing the following: (a) diffuse positivity for S-100 protein (×200), (b) desmin positivity in the rhabdomyoblast cells (×200), and (c) myoglobin positivity in the rhabdomyoblast cells (×400).

Macroscopically, the tumor was oval in shape, partially encapsulated with a smooth yellowish-white surface. The cut surface showed microcysts, hemorrhage, and necrosis with brownish areas (Figure 3). On microscopic examination. A partially encapsulated cellular mass composed of interlacing fascicles of hyperchromatic serpentine spindle cells was found. The cells had elongated and comma shaped nuclei with prominent nucleoli and sparse cytoplasm with indistinct borders. Large pleomorphic cells with abundant eosinophilic cytoplasm and rounded eccentric nuclei with large nucleoli were present (Figures 4(a), 4(b), and 4(c)). Some of these identified cells were striated, strap-like, or globoid rhabdomyoblasts. These cells were identified on hematoxylin and eosin and phosphotungstic acid hematoxylin stains (Figure 4(d)). Necrosis was limited to few areas. The surgical margins were free of disease. Immunohistochemical staining (IHS) of the tumor revealed diffuse, intense positivity of the wavy spindle cells for S-100 protein, confirming its neurogenic origin (Figure 5(a)). Myogenic differentiation of the large pleomorphic cells was confirmed by the strong positivity for desmin (Figure 5(b)) and moderate positivity for myoglobin (Figure 5(c)). The microscopic examination and the reaction to the previous markers confirmed the diagnosis of a low grade MTT.

The annual follow-up CT scans revealed no evidence of recurrence or metastases. Our patient is alive and still free of recurrences four years after initial diagnosis.

## 3. Discussion

A so-called mosaic tumor with both muscular and neurogenic components. In spite of being a rare tumor, the head and neck region is one of the most frequent sites of involvement of MTT. For this reason, head and neck surgeons and pathologists should be familiar with the nature, the treatment modalities, and prognosis of this rare tumor [21, 22].

Our reported case in the neck was sporadic. It seems that the head and neck neoplasms are infrequently associated with NF-1 compared to those located in other sites [22]. Our patient's age falls within the average age of 30 years reported in the literature [23]. However, a wide age range from newborns to extremely older individuals has been reported by others [24, 25].

In the present case, the IHS was essential to reach the proper diagnosis. Tumor morphology of a malignant schwannoma with potentially overlooking the neoplastic rhabdomyoblasts makes IHS essential in the diagnosis of MTT. However, Weiss and Enzinger [26] mentioned that discrete rhabdomyoblastic differentiation is necessary to label a MTT. The considerable difficulty to diagnose this tumor is attributed to its microscopic similarity to a wide variety of sarcomas [27]. Therefore, IHS is important. This is in accordance with other investigators [23, 27–29]. Furthermore, it was found that electron microscopic study can be used in the diagnosis of these tumors, although not performed in the present case [3, 27, 29].

Once the diagnosis was done, complete tumor resection with negative margins should be the goal of surgery, as in the present case. Therefore, it would certainly make sense to consider the use of such treatment without delay. This is consistent with other researchers [21, 30, 31].

The visibility of the neck mass in our reported case made its timely diagnosis possible. Reports of tumors in noticeable sites aid in the it's early diagnosis and excision while the tumor is still small leading to a better prognosis. Such areas include the extremities [18] and the head and neck [21, 30, 31]. This is in contrast to the findings of other investigators [12–14].

The size of the tumor is also an important prognostic factor affecting survival. In the current case, the tumor size was smaller than 10 cm. It was mentioned that patients with tumors less than 10 cm had better survival than those with larger tumors [3, 18, 32]. This could probably be related to the stage of the tumor at the time of presentation.

Victoria et al. [28] reviewed the treatment and outcome of 27 MTTs arising in the head and neck and commented that there may be a subset of MTT occurring in this region as a low-grade malignancy with favorable long-term prognosis. Their findings were supported by other researchers [30, 31]. Unlike the previous opinions, it was reported that rhabdomyoblastic differentiation within the tumor is fatal and causes it to behave aggressively [2, 4, 12–14].

Furthermore, our case was sporadic and of low-grade malignancy. This is in accordance with Brooks et al. [25] who reported that when MTT is not associated with NF-1, it is usually of low grade.

The clinical, microscopic, and long-term follow-up of the present case showed that our case was a low-grade malignancy. Finally, we would like to emphasize that early diagnosis and wide surgical treatment are important for the prognosis of MTT.
